# Supplementary material for: Bosutinib Stimulates Macrophage Survival, Phagocytosis, and Intracellular Killing of Bacteria
Source: ACS Infect Dis. 2024 Apr 11;10(5):1725–38. doi: 10.1021/acsinfecdis.4c00086 (PMC11091880; doi:10.1021/acsinfecdis.4c00086)
Supplement: Supplementary file 1 — id4c00086_si_001.pdf [file id4c00086_si_001.pdf]

Supporting Information

Bosutinib stimulates macrophage survival, phagocytosis and intracellular killing of bacteria

Ronni A. G. da Silva[1,2], Claudia J. Stocks[2], Guangan Hu[3], Kimberly A. Kline[1,2,4 *], Jianzhu Chen[1,3 *]

1- Singapore-MIT Alliance for Research and Technology, Antimicrobial Drug Resistance Interdisciplinary Research Group, 138602, Singapore.

2- Singapore Centre for Environmental Life Sciences Engineering, Nanyang Technological University, 637551, Singapore.

3- Koch Institute for Integrative Cancer Research and Department of Biology, Massachusetts Institute of Technology, Cambridge, MA, 02139, USA.

4- Department of Microbiology and Molecular Medicine, Faculty of Medicine, University of Geneva, Geneva, 1211, Switzerland.

* kimberly.kline@unige.ch

* jchen@mit.edu

Pages: 14; Supplementary Figures: 7; Supplementary Tables: 4; Additional References: 4

Supplementary Figures

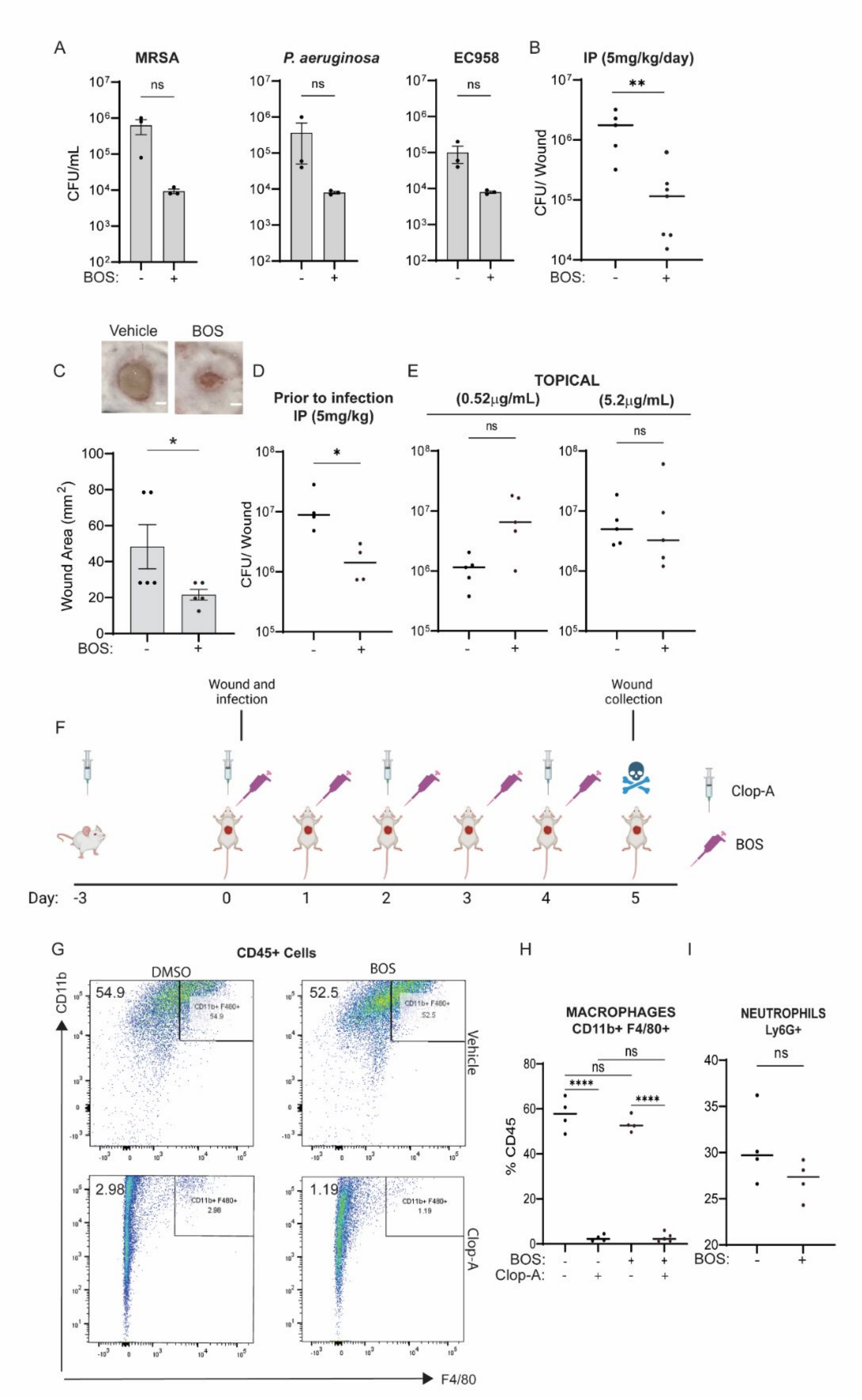

Figure S1- Macrophages are required for BOS-induced phenotype *in vivo*. (A) Comparison of MRSA, *P. aeruginosa* and *E. coli* EC958 CFU in RAW264.7 in the

presence or absence of BOS (0.52 μg/mL). **(B)** Comparison of VRE CFU per infected wound of animals treated with five IP injections of vehicle or BOS (30 μL of 5mg/kg). **(C)** Representative images of wounds (top panel) and summary of data from five mice (low panel) at the end of the multiple-treatment experiment. Scale bars, 2 mm. Wound area measured at 4 dpi after five treatments. **(D)** Comparison of VRE CFU per infected wound of animals treated with a single IP injection of vehicle or BOS (5mg/kg) 24h prior to infection. **(E)** Comparison of VRE CFU per infected wound treated topically a single dose of vehicle or BOS (5.20 μg/mL). **(F-I)** A schematic diagram of experimental design (F). Mice were injected IP with clop-A (200 μL, 6 mg/mL) 3 days prior to wounding and infection, and additional doses of clop-A on the day of wounding and infection and every 2 days afterwards. In addition, clop-A (10 μL, 6 mg/mL) was applied to the wounds every 2 days. Following VRE infection, BOS (10μL, 0.52 μg/ml) was applied to the wounds daily for 5 days. Five days after wounding and infection, mice were sacrificed, and wounds were recovered for assaying macrophage depletion and VRE CFU. Representative flow cytometry of macrophages ($CD45^+$ $CD11b^+$ $F4/80^+$) from infected wounds (G). The number indicate percentages of cells within the gated areas. Comparison of the percentages of macrophages recovered from infected wounds with or without clop-A and/or BOS treatments (H). Comparison of the percentages of neutrophils recovered from infected wounds that were vehicle or BOS treated with five topical doses (I). Each symbol represents one mouse with the median indicated by the horizontal line (B, D-E, and H-I). Data were from at least two independent experiments with two to three mice per experiment. Statistical analysis was performed using unpaired t test with Welch's corrections (A), using the nonparametric Mann-Whitney test to compare ranks or using Kruskal-Wallis test with uncorrected Dunn's posttest (B-E, H-I)). NS, $P > 0.05$; $^*P \le 0.05$, $^{**}P \le 0.01$, $^{***}P \le 0.001$ and $^{****}P \le 0.0001$.

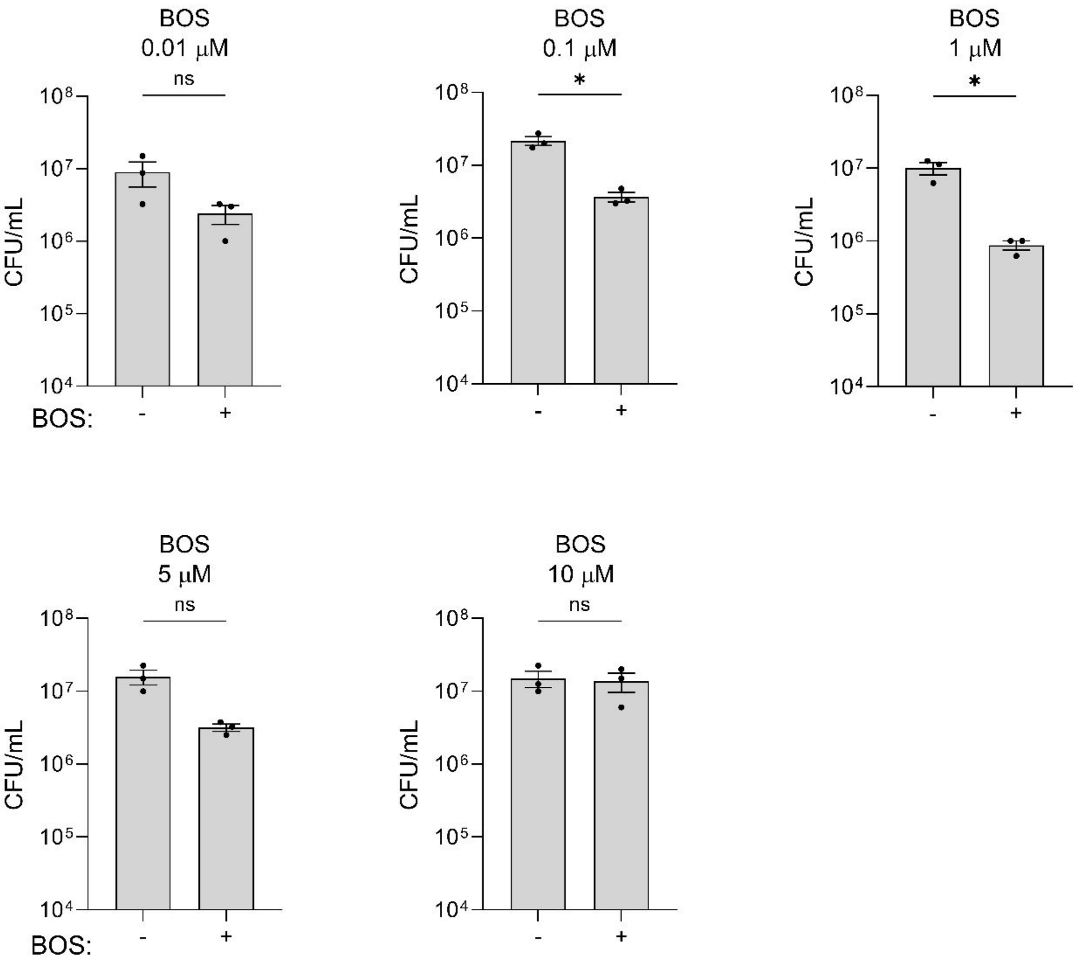

Figure S2- Different doses of BOS enhance macrophage killing of intracellular bacteria *in vitro*. Comparison of VRE CFU in RAW264.7 cells treated with BOS (0.01 - 10 μM) for 15 h after initial infection of 3 h. Statistical analysis was performed using unpaired t test with Welch's corrections. NS denotes not significant; and *P ≤ 0.05.

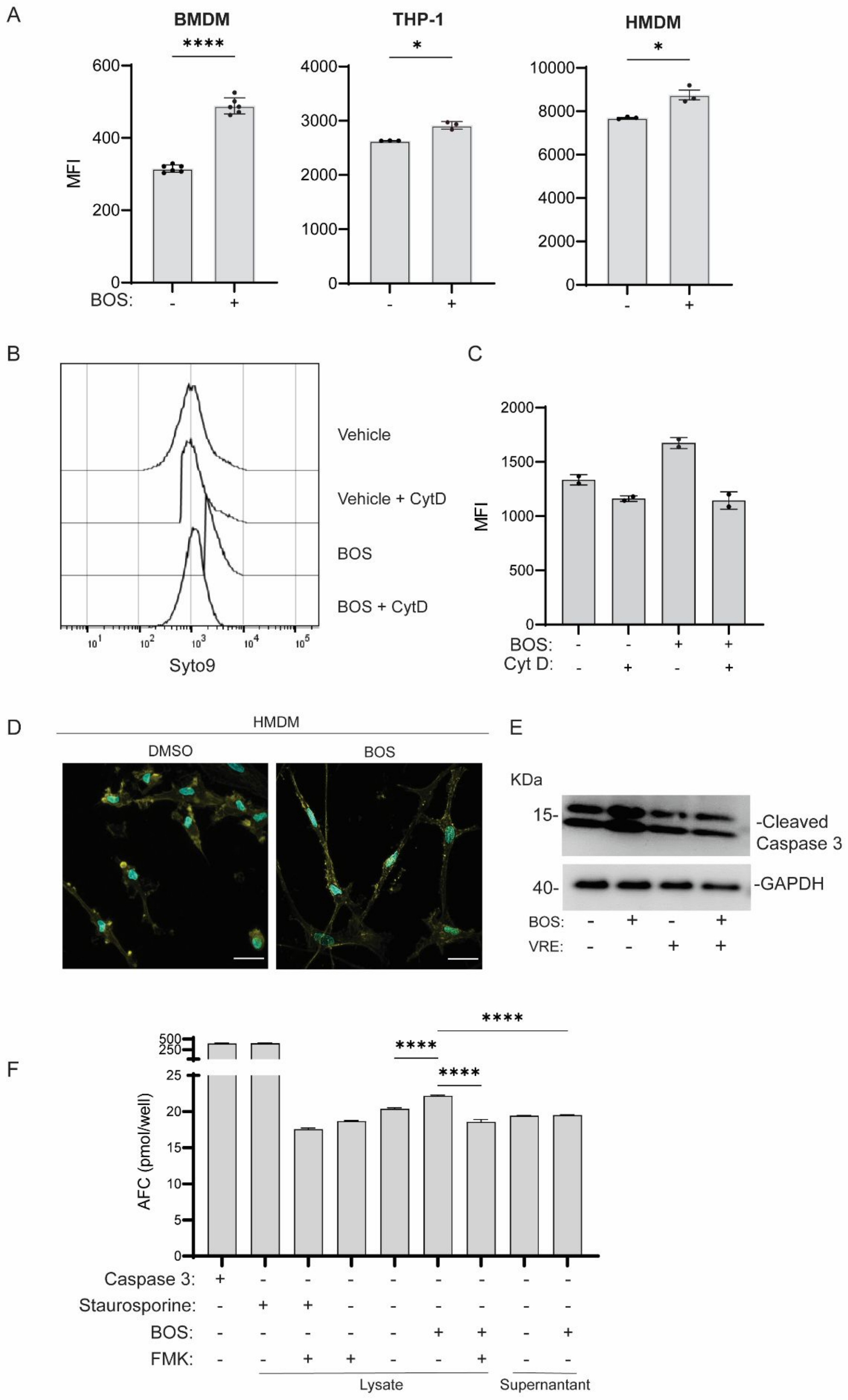

Figure S3- BOS stimulates macrophage phagocytosis. (A) Phagocytosis of VRE by BMDM, THP-1 and HMDM in the presence or absence of BOS. Data (mean ± SEM)

are summary of at least three independent experiments. **(B-C)** Comparison of uptake of SYTO9-labelled VRE by RAW264.7 macrophages in the presence or absence of BOS or CytD. RAW264.7 macrophages with and without BOS pre-treatment in the presence or absence of CytD were infected for 1h with SYTO9-labelled VRE, followed by quenching with trypan blue and measurement of fluorescence intensity by flow cytometry. Shown are the representative staining profiles (B) and MFI from two independent experiments (C). **(D)** Representative CLSM images of DMSO or BOS treated HMDM samples that were stained with phalloidin for actin visualization and Hoechst 33342 for nucleus visualization. Images are maximum intensity projections of the optical sections (0.64 µm z-volume) and are representative of 3 independent experiments. Scale bar: 20 µm. **(E)** Western blotting analysis of cleaved caspase 3 in RAW264.7 cells in response to BOS treatment and VRE infection. Whole cell lysate was Western blotted with anti-cleaved caspase 3 and anti-GAPDH. **(F)** Caspase 3 activity assay of cell lysates or supernatants of RAW234.7 cells that were non-treated or treated with BOS. Staurosporine (100 µM) and FMK (50 µM) were also included as positive and negative controls for intracellular Caspase 3 activation, respectively. Data (mean ± SEM) are a summary of at least three independent experiments. Statistical analysis was performed using unpaired t test with Welch's corrections (A, C) or ordinary one-way ANOVA, followed by Tukey's multiple comparison test (F). *$P \leq 0.05$, and ****$P \leq 0.0001$.

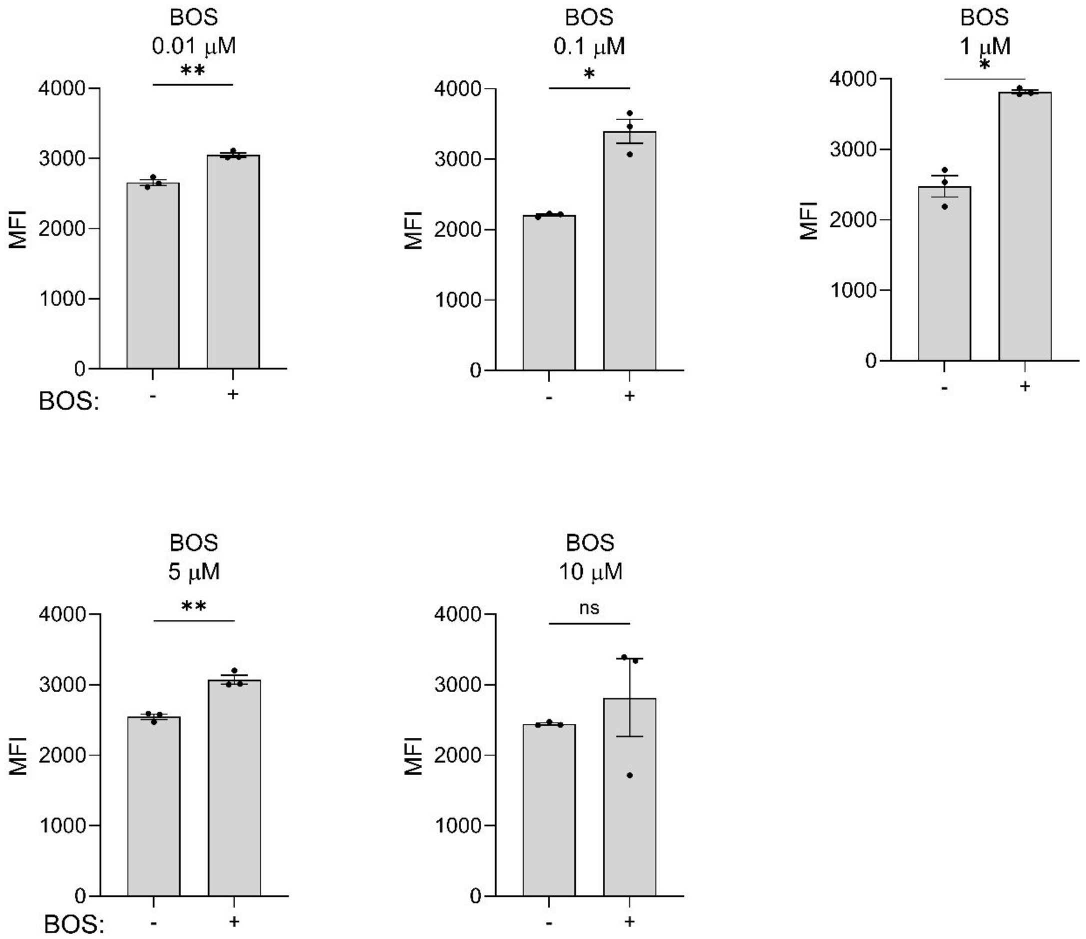

Figure S4- BOS stimulates macrophage phagocytosis across a range of doses *in vitro*. Comparison of the uptake of SYTO9-labelled VRE by RAW264.7 macrophages in the presence or absence of BOS with concentrations varying from 0.01 to 10 μM. RAW264.7 macrophages with and without BOS pre-treatment were infected for 1h with SYTO9-labelled VRE, followed by quenching with trypan blue and measurement of fluorescence intensity by flow cytometry. Shown are the MFI from three independent experiments. Statistical analysis was performed using unpaired t test with Welch's corrections. NS denotes not significant; *P ≤ 0.05; and **P ≤ 0.01.

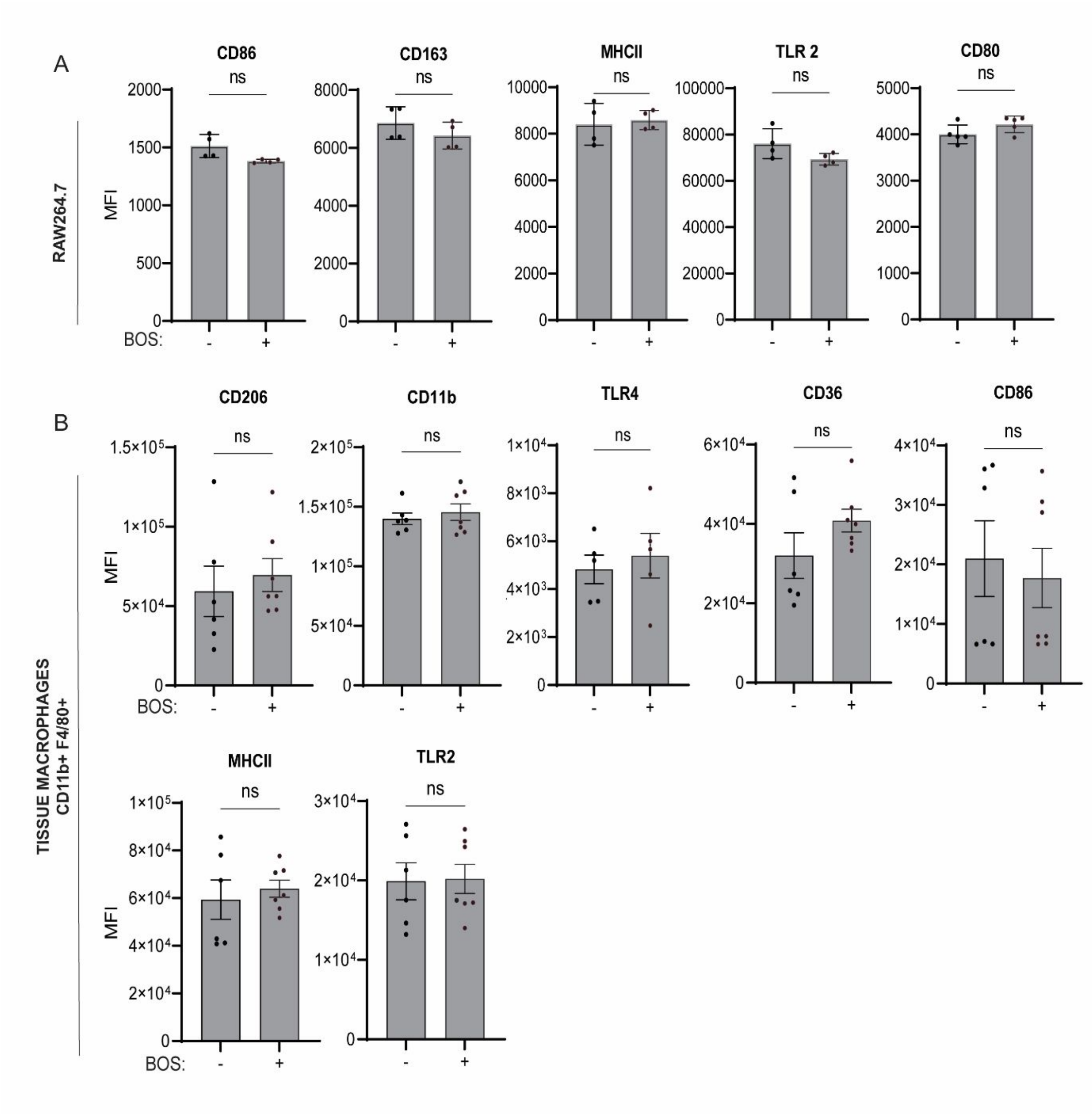

Figure S5- Effect of BOS treatment on the expression of surface markers related to bacterial uptake. (A-B) Comparison of MFI of bacterial recognition, uptake and presentation surface markers gating on $CD45^+$ RAW264.7 macrophages non-treated or treated with BOS (A) and $CD45^+$ $CD11b^+$ $F4/80^+$ macrophages from wounds of animals treated with an IP injection of vehicle (-) or BOS (+) (B). Data (mean ± SEM) are summary of at least two independent experiments (A-B) with two to four mice per experiment. Statistical analysis was performed using unpaired t test with Welch's corrections. NS, $P > 0.05$; *$P \leq 0.05$, **$P \leq 0.01$, ***$P \leq 0.001$, and ****$P \leq 0.0001$.

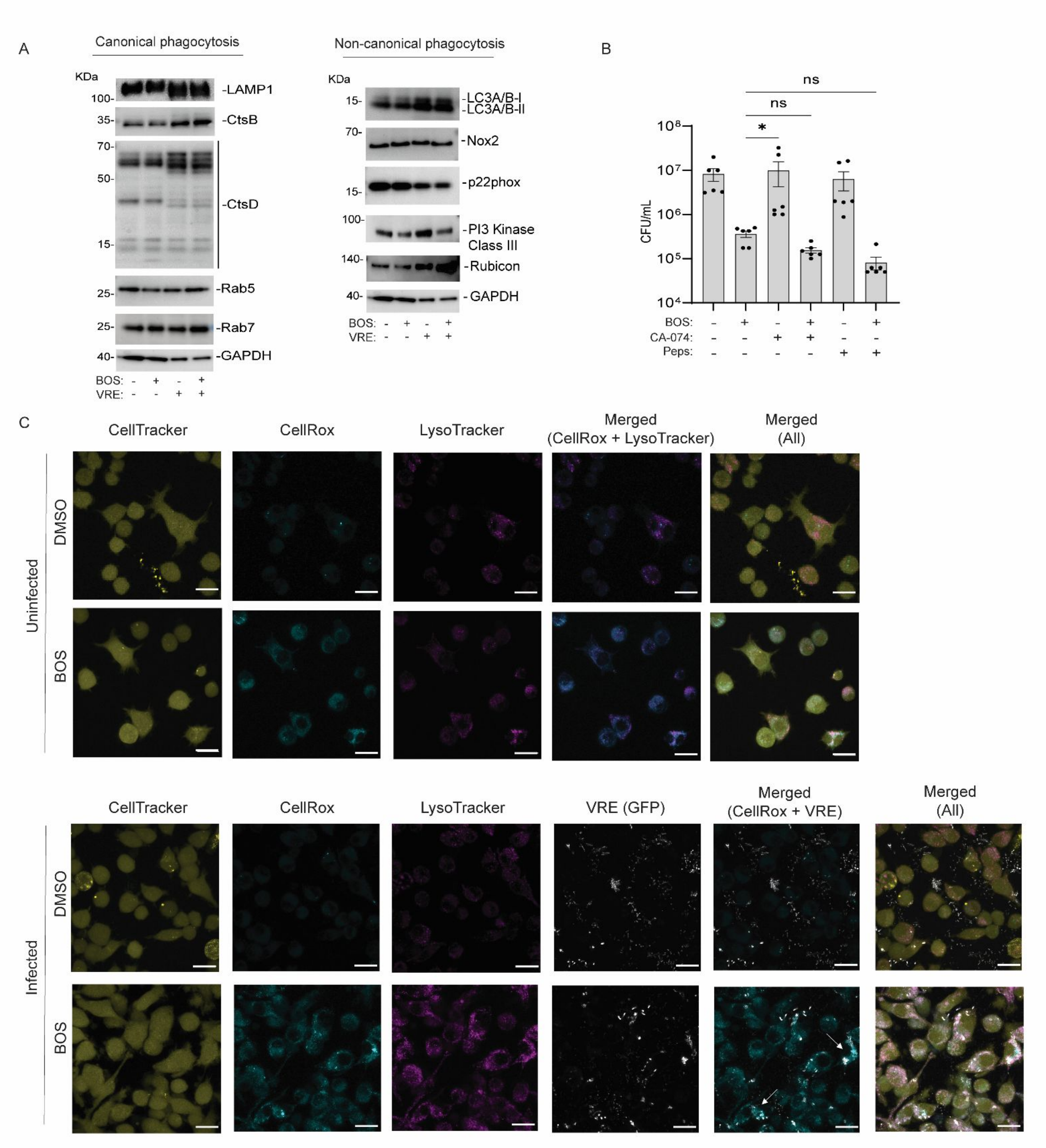

Figure S6- BOS-treated macrophages produce more ROS. (A) Western blot of whole-cell lysates for proteins of the canonical and non-canonical phagocytosis pathway. RAW264.7 cells with (+) and without (−) VRE infection were treated with BOS (+) or left untreated (−). Cell lysates were separated by SDS-PAGE and the

proteins were transferred to a membrane. The same membrane was Western blotted with various antibodies and anti-GAPDH. Shown are representative data from at least two independent experiments. **(B)** Effect of cathepsin inhibitors on BOS-stimulated bacterial killing by macrophages. RAW264.7 cells were infected with VRE in the presence of BOS (0.52 μg/mL), CtsB inhibitor CA-074 (5 nM), and CtsD inhibitor Pepstatin A (Peps, 10 μg/mL) alone or in combination. Intracellular bacterial CFU was quantified after 18h. Data (mean ± SEM) are a summary of at least three independent experiments. Statistical analysis was performed using ordinary one-way ANOVA, followed by Tukey's multiple comparison test. NS, $P > 0.05$; *$P \leq 0.05$, **$P \leq 0.01$, and ****$P \leq 0.0001$. **(C)** Visualization of ROS in RAW264.7 cells following BOS and VRE infection by microscopy. Representative CLSM images of DMSO or BOS-treated RAW264.7 samples that were stained with CellTracker (yellow) for cell shape visualization, CellRox (cyan) for ROS visualization and LysoTracker (magenta) for lysosomes visualization. Bottom panels were also infected with pDasher GFP-expressing VRE cells (gray). White arrows point to areas with intracellular VRE cells and high levels of ROS. Images are maximum intensity projections of the optical sections (0.64 μm z-volume) and are representative of at least 2 independent experiments. Scale bar: 20 μm.

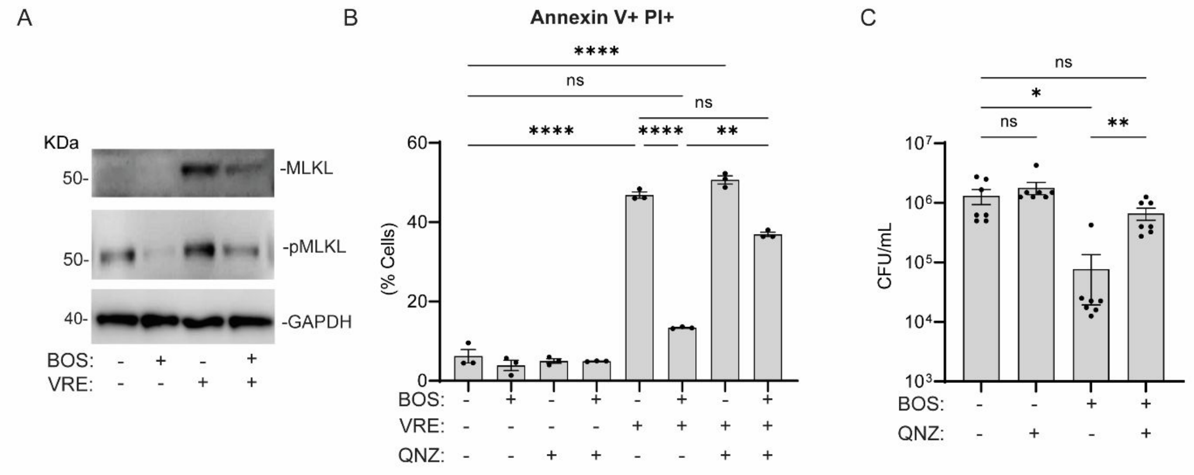

Figure S7- BOS promotes survival of infected macrophages. (A) Western blotting analysis of MLKL and pMLKL. RAW264.7 cells with (+) and without (−) VRE infection were treated with BOS (+) or left untreated (−). Whole-cell lysates were Western blotted with anti-MLKL, anti-pMLKL and anti-GAPDH antibodies. **(B)** Comparison of percentage of Annexin V^+ and PI^+ cells at the end of infection. RAW264.7 cells were either infected or not infected and were treated with BOS alone or in combination with the NF-κB inhibitor QNZ (10 nM). Annexin V and PI reactivity was assayed by flow cytometry. Data (mean ± SEM) are a summary of at least three independent experiments. **(C)** RAW264.7 cells were infected with VRE in the presence of BOS (0.52 μg/mL), and QNZ (1 nM), alone or in combination. Intracellular bacterial CFU was quantified after 18 h. Statistical analysis was performed using ordinary one-way ANOVA, followed by Tukey's multiple comparison test (B), or Brown-Forsythe and Welsh ANOVA test (C). NS, P > 0.05; *P ≤ 0.05, **P ≤ 0.01, and ****P ≤ 0.0001.

Supplementary Tables

Table S1- Bacterial strains used in this study.

Strain	Reference
E. faecalis **V583 (VRE)**	1
E. faecalis V583 **+ pDasher GFP**	This study
S. aureus **USA300 (MRSA)**	2
E. coli **EC958**	3
P. aeruginosa **PAO1**	4

Table S2- MIC of BOS and antibiotics alone or in combination with 0.52 µg/mL BOS.

Compound or Antibiotic	MIC (µg/mL)	MIC in combination with BOS (0.52 µg/mL)	Strain
BOS	>13	-	VRE
Vancomycin	18	18	VRE
Penicillin G	2	2	VRE
Gentamycin	1000	500	VRE
BOS	>13	-	MRSA
BOS	>13	-	*P. aeruginosa* PAO1
BOS	>13	-	*E. coli* EC958

Table S3 – Cytotoxicity as measured by LDH assay of compounds used in this study.

Condition	% Cytotoxicity ±SD	Source
DMEM (Baseline)	8.72 ±4.67	Gibco
BOS (1 μM)	12.38 ±1.31	Sigma
BOS (5 μM)	12.93 ±2.86	Sigma
BOS (10 μM)	28.69 ±1.16	Sigma
BOS (50 μM)	64.66 ±1.58	Sigma
Cytochalasin D (40 μM)	-	Abcam
QNZ (10 nM)	18.62 ±13.44	Abcam
CA-074 (5 nM)	-	MedChemExpress
Pepstatin A (10 μg/mL)	-	Sigma
TEMPO (50 μM)	13.77 ±2.37	Sigma
SLK (1 μM)	1.56 ±3.18	MedChemExpress
BOS (1 μM) + SLK (1 μM)	4.38 ±0.99	-
FMK (50 μM)	-	Abcam
BOS (1 μM) + FMK (50 μM)	-	-
DMAT (1 μM)	15.44 ±4.30	MedChemExpress
SARA (1 μM)	-	MedChemExpress
DASA (1 μM)	10.81 ±10.73	MedChemExpress
TIR (0.33 μM)	13.41 ±6.64	MedChemExpress

Table S4- Comparison of transcript levels of cell surface markers associated with bacterial recognition, uptake, and presentation in RAW264.7 cells with and without BOS treatment.

Gene	logFC	pValue
CD36	-2.5004664	1.31E-12
CD80	1.06914694	3.32E-06
CLEC7A (DECTIN-1)	-0.1803542	2.79E-01
CD14	-0.2248473	1.39E-01
ITGAM (CD11B)	0.74697971	8.90E-07
TLR4	0.48315495	2.53E-04
TLR2	-0.0713851	5.92E-01
TLR8	1.11318224	8.89E-12

Additional References:

1. Bourgogne A, Garsin DA, Qin X, Singh KV, Sillanpaa J, Yerrapragada S, … Weinstock GM. Large scale variation in Enterococcus faecalis illustrated by the genome analysis of strain OG1RF. Genome Biol. 2008;9(7):R110.

2. McDougal LK, Steward CD, Killgore GE, Chaitram JM, McAllister SK, Tenover FC. Pulsed-field gel electrophoresis typing of oxacillin-resistant Staphylococcus aureus isolates from the United States: establishing a national database. J Clin Microbiol. 2003;41(11):5113-20.

3. Totsika M, Beatson SA, Sarkar S, Phan MD, Petty NK, Bachmann N, ... Schembri MA. Insights into a multidrug resistant Escherichia coli pathogen of the globally disseminated ST131 lineage: genome analysis and virulence mechanisms. PLoS One. 2011;6(10):e26578.

4. Hentzer M, Riedel K, Rasmussen TB, Heydorn A, Andersen JB, Parsek MR, … Givskov M. Inhibition of quorum sensing in Pseudomonas aeruginosa biofilm bacteria by a halogenated furanone compound. Microbiology (Reading). 2002;148(Pt 1):87-102
